# PD-1/PD-L1 Axis as a Potential Therapeutic Target for Multiple Sclerosis: A T Cell Perspective

**DOI:** 10.3389/fncel.2021.716747

**Published:** 2021-07-26

**Authors:** HaiXia Li, Chao Zheng, Jinming Han, Jie Zhu, Shan Liu, Tao Jin

**Affiliations:** ^1^Department of Neurology and Neuroscience Center, The First Hospital of Jilin University, Changchun, China; ^2^Department of Clinical Neuroscience, Karolinska Institutet, Solna, Sweden; ^3^Department of Neurology, Xuanwu Hospital, Capital Medical University, Beijing, China; ^4^Department of Neurobiology, Care Sciences and Society, Karolinska Institutet, Solna, Sweden

**Keywords:** experimental autoimmune encephalomyelitis, multiple sclerosis, T cells, programmed cell death ligand-1, programmed cell death protein-1

## Abstract

The programmed cell death protein-1/programmed death ligand-1 (PD-1/PD-L1) axis is a widely studied immune checkpoint that modulates signaling pathways related to T cell activation. The use of PD-1/PD-L1 inhibitors is a promising immune therapy strategy for cancer patients. However, individuals treated with PD-1/PD-L1 inhibitors may develop immune-related adverse events due to excessive immune reactions. Multiple sclerosis (MS) is a chronic demyelinating and neurodegenerative disease of the central nervous system. T cells and the PD-1/PD-L1 axis play vital roles in the pathogenesis of MS. A better understanding of the complex relationship between the PD-1/PD-L1 axis and T cells may extend our knowledge of the molecular mechanisms and therapeutic approaches for MS. In this review, we summarize the most recent findings regarding the role of the PD-1/PD-L1 axis in MS and discuss the potential therapeutic strategies to modulate the expression of PD-1/PD-L1 in MS.

## Introduction

Multiple sclerosis (MS) is an autoimmune disease characterized by demyelination, axonal injury, and neuronal loss in the central nervous system (CNS), causing a variety of clinical symptoms involving motor, sensory, visual, and autonomic systems (Dobson and Giovannoni, 2019). As a leading non-traumatic disabling disease among young adults, MS affects more than 2 million people worldwide and causes considerable social and economic burdens (Thompson et al., 2018). Experimental autoimmune encephalomyelitis (EAE) is a widely used animal model of MS (Glatigny and Bettelli, 2018). Accumulating evidence indicates that MS and EAE are mediated by activated T cells, B cells, and innate immune cells (Cheng et al., 2017; Yang et al., 2019). However, the pathogenesis of MS is complex and more efforts are needed to elucidate the mechanisms underlying this process.

The inhibitory receptor programmed cell death protein-1 (PD-1) and its ligand programmed death ligand-1 (PD-L1) are negative regulators of immune responses, which maintain immune tolerance by regulating the expansion, differentiation, and activation of immune cells (Schreiner et al., 2008; Ding et al., 2016). A growing number of studies indicate that the PD-1/PD-L1 axis, as a brake on T cell activation, participates in the pathogenesis of autoimmune diseases, including systemic lupus erythematosus (SLE) (Prokunina et al., 2002), rheumatoid arthritis (RA) (Kong et al., 2005), type 1 diabetes (T1D) (Wang et al., 2005), autoimmune hepatitis (AIH) (Curran and Sharon, 2017), and MS (Kroner et al., 2005). In this article, we mainly focus on the involvement of the PD-1/PD-L1 axis in MS/EAE. Novel insights into the modification of the PD-1/PD-L1 axis as a therapeutic strategy for MS have been highlighted.

## Overview of PD-1/PD-L1

PD-1 and PD-L1 play an essential role in maintaining immune homeostasis (Keir et al., 2006). Ishida et al. (1992) first discovered that the immune checkpoint gene *PD-1* was significantly upregulated during the generation of murine T cell hybridoma. The activation of the *PD-1* gene is involved in programmed cell death and can promote the evasion of tumor cells from effective immune responses. By contrast, checkpoint inhibitors restore the anti-cancer activity of T cells (Ishida et al., 1992). PD-1 is a member of the B7-CD28 superfamily of type I transmembrane protein receptors with 288 amino acids, encoded by the *PDCD1* gene and is located on chromosome 2q37.3. It is mainly expressed on the surface of activated T cells, B cells, dendritic cells (DC), monocytes, and natural killer cells (Chen, 2004; Okazaki and Honjo, 2006; Keir et al., 2008; Xia et al., 2016). PD-L1 (also known as B7-H1) and PD-L2 (also known as B7-DC) are two ligands of PD-1, both located on chromosome 9p21.4 and are encoded by *CD274* and *PDCD1LG2*, respectively (Xia et al., 2016). PD-L1 is mainly expressed on activated T cells, B cells, DCs, macrophages, mesenchymal stem cells, and cultured bone marrow-derived mast cells, whereas PD-L2 is mainly expressed on DCs, macrophages, and cultured bone marrow-derived mast cells (Augello et al., 2005; Nakae et al., 2006). The expression of PD-1 and its binding affinity to ligands regulate the threshold of T cell tolerance induction and maintenance, as well as immune cell activation and cytokine secretion (Sharpe et al., 2007). The PD-1/PD-L1 axis regulates immune tolerance and its dysfunction leads to a variety of autoimmune diseases (Zamani et al., 2016).

## Role of PD-1 in T Cells

**FIGURE 1:**
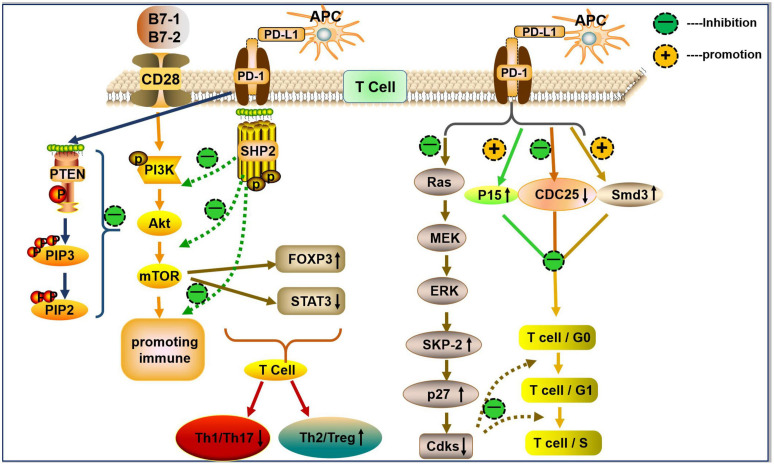
The PD-1/PD-L1 axis in T cells differentiation and proliferation. PD-L1 (mainly expressed on APCs) binds to PD-1 on the surface of T cells and generates a co-inhibitory signal. The PD-1/PD-L1 interaction recruits SHP2 to block CD28-mediated activation of PI3K and AKT (i.e., reduce the levels of p-P13K and p-AKT). PD-L1 decreases the activity of mTOR by inhibiting p-AKT, and the mTOR pathway upregulates the expression of Foxp3 (↑) and downregulates the expression of p-STAT3 (↓), thereby inducing Treg/Th2 differentiation and inhibits Th1/Th17 cell differentiation. PD-L1 can promote the expression of PTEN. PTEN dephosphorylates the inositol PIP_3_ on cell membranes to generate PIP_2_ and then antagonizes PI3K, which mediates cell growth, metabolism, proliferation, and survival signals. The PD-1/PD-L1 interaction inhibits Ras, which inhibits the activation of downstream MAPK/ERK signaling, leading to a reduction in ubiquitin ligase SCF^*Skp*2^-driven degradation of p27^*kip*1^. This process results in the accumulation of p27^*kip*1^, inhibition of Cdks, and further inhibition of T cell proliferation. Furthermore, PD-1 inhibits T cell proliferation by upregulating p27, p15, and Smad3, but inhibiting CDC25A.

The cytoplasmic tail of PD-1 contains two tyrosine motifs: an immunoreceptor tyrosine-based inhibition motif (ITIM) and an immunoreceptor tyrosine-based switch motif (ITSM) (Okazaki et al., 2001). Upon PD-L1 engagement, ITIM and ITSM are phosphorylated and induce the recruitment of phosphatase Src homology-2 domain-containing protein tyrosine phosphatase (SHP2) in T cells (Okazaki et al., 2001; Sheppard et al., 2004). Subsequently, phosphorylated ITIM and ITSM reduce the phosphorylation of CD3 ζ chain, ZAP70 tyrosine, and protein kinase C (PKC)-θ, downregulate the phospha-tidylinositol-3-kinase (PI3K) and serine/threonine-protein kinase (AKT) signaling pathway, and inhibit T cell proliferation and cytokine production (Sheppard et al., 2004; Hofmeyer et al., 2011). Moreover, PD-1 recruits SHP2 through the ITSM to block CD28-mediated activation of the PI3K/AKT/mTOR signaling (Parry et al., 2005). The inactivation of AKT blocks cell cycle progression by activating pro-apoptotic factors, such as Bad and procaspase-9. It also inhibits protein synthesis and glycogen metabolism by regulating mTOR, glycogen synthase kinase 3, insulin receptor substrate 1, cyclin-dependent kinase inhibitors p21^*CIP*1/WAF1^ and p27^*KIP*1^, and RAF-1 to block cell cycle progression and cell growth (Porta et al., 2014). PD-1/PD-L1 have also been shown to increase the expression of phosphatase and tensin homolog (PTEN) (Francisco et al., 2009). PTEN dephosphorylates phosphatidylinositol (3,4,5)-triphosphate (PIP3) on the cell membrane and generates phosphatidylinositol 4,5-bisphosphate (PIP2), antagonizing both the PI3K/AKT and mitogen-activated protein kinase (MAPK)/extracellular-signal-regulated kinase (ERK) signaling pathways, thereby mediating cellular metabolism, proliferation, and survival (Weng et al., 2001; Francisco et al., 2009; Laplante and Sabatini, 2012; Figure 1).

## Regulation of T Cells Function

**FIGURE 2:**
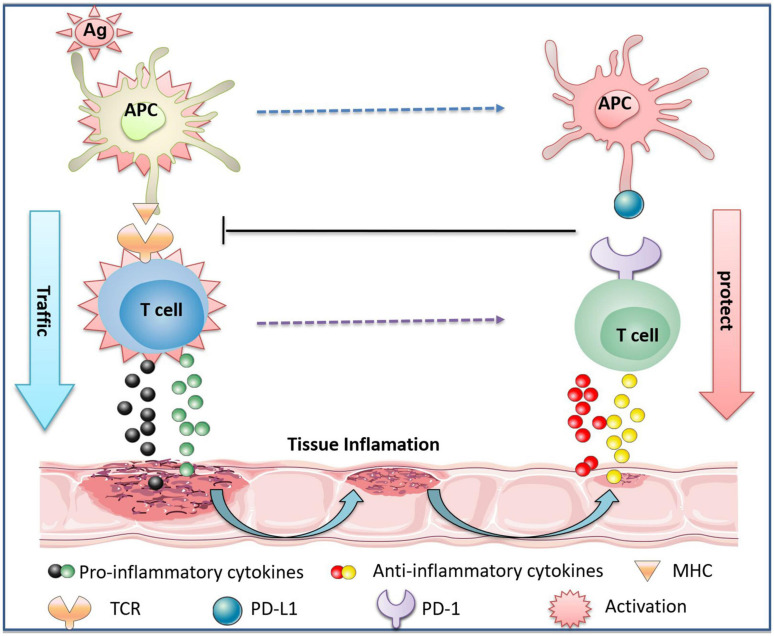
The protective effect of PD-1/PD-L1 on immune inflammatory tissues through T cells. The antigen (Ag) invades the body to activate APC. It is captured by major histocompatibility complex (MHC) molecules on APC, which binds to TCR on T cells to activate T cells. Activated T cells release pro-inflammatory cytokines, causing tissue damage. In parallel, activated APC and T cells express PD-L1 and PD-1, respectively, and the interaction of PD-L1 and PD-1 promotes the release of anti-inflammatory cytokines by T cells. The binding of PD-L1 to PD-1 also inhibits the activation of APC and T cells, thereby reducing tissue inflammation, promoting tissue repair, and protecting normal tissues from immune inflammatory response-induced damage.

PD-1 is located adjacent to the Golgi compartments in T cells and its expression is tightly regulated (Pentcheva-Hoang et al., 2007). The level of PD-1 in naïve T lymphocytes is low or even undetectable. However, the expression of PD-1 can be rapidly induced when the T cell receptor (TCR, located on T cells) is activated. TCR activation transduces extracellular signals into cellular responses and activates specific downstream signaling, such as MAPK, PKC, calcium, etc., which ultimately causes T cell proliferation, activation, and differentiation (Pentcheva-Hoang et al., 2007; Garcia et al., 2009; Smith-Garvin et al., 2009). PD-1/PD-L1 deliver co-suppressive signals to TCR, subsequently decreasing the activation of autoreactive T cells (Bennett et al., 2003; Brown et al., 2003). The co-stimulatory molecule CD28 expressed on the surface of T cells binds to the B7 on antigen-presenting cells (APC), mediating T cell costimulation and promoting proinflammatory cytokine production (Esensten et al., 2016). PD-1 blocks the activation of the above signals when bound to PD-L1, thereby limiting inflammatory responses associated with peripheral T cell activity (Ansari et al., 2003; Keir et al., 2006; Figure 2). In addition, PD-1/PD-L1-deficient mice exhibited a low threshold of TCR stimulation to activate T cells and were prone to inflammation, such as lupus-like proliferative arthritis and glomerulonephritis (Nishimura et al., 1999; Freeman et al., 2000).

Previous studies have shown that the transcription efficiency of TCR-dependent genes is related to their sensitivity to PD-1-mediated downregulation. The genes that encode survival and proliferation-related proteins (i.e., *Nfatc1, Nrf1, Egr1, Nr4a1, Cdc14b, Erbb3*, etc.) are not sensitive to PD-1, and are therefore highly expressed after TCR activation. By contrast, the genes encoding cytokines and effector molecules (i.e., *Ifng, Il3, Il4, Tnf, Egr2*, etc.) are PD-1-sensitive genes and can therefore be effectively inhibited by PD-1 (Shimizu et al., 2020). These findings indicate that the main role of PD-1 in activating T cells is to inhibit the secretion of cytokines and effector molecules.

## PD-1/PD-L1 in MS/EAE

Ortler et al. demonstrated for the first time that B7-H1 was highly expressed in the inflammatory areas of white matter in MS patients and was likely to be expressed on activated microglia/macrophage, which was consistent with the hypothesis that B7-H1 expression may reduce T cell activation and promote immune homeostasis in the CNS (Ortler et al., 2008). The polymorphism of the *PD-1* gene is considered a modifier of MS progression. In MS, PD-1-mediated inhibition of interferon (IFN)-γ by T cells can be damaged in patients carrying *PD-1* gene polymorphism (Kroner et al., 2005). The number of PD-1^+^CD4^+^ T cells, PD-1^+^CD8^+^ T cells, PD-L1^+^ interleukin (IL)-10^+^CD14^+^ cells, and PD-L1^+^IL-10^+^CD19^+^ cells in MS patients are significantly increased during the remitting phase compared with the relapsing phase, indicating that PD-1/PD-L1 also plays an immunosuppressive role in MS. The inhibition of PD-1 in lymphocytes in the acute phase of MS significantly increases the proliferation of CD4^+^ T cells and CD8^+^ T cells. However, no such effect is observed when the expression of PD-L1 is blocked in APC, indicating that PD-1 is more dominant than PD-L1 in promoting lymphocyte apoptosis and inhibiting proliferation in MS (Trabattoni et al., 2009). In addition, the expression of PD-1/PD-L1, but not PD-L2, on peripheral blood mononuclear cells (PBMC) was significantly downregulated in patients with MS compared with healthy controls (Javan et al., 2016). Although the regulatory mechanisms of the PD-1/PD-L1 axis during MS onset and progression remain unclear, it has been gradually established that the PD-1/PD-L1 axis is a classical immune suppressor in MS (Javan et al., 2016). PD-1/PD-L1 is highly expressed on activated immune cells. The expression of PD-1/PD-L1 in different immune cells in MS requires further investigation with a large sample size.

The PD-1/PD-L1 signaling also regulates the pathogenesis of EAE. The blockade of PD-1 in EAE resulted in the activation of antigen-specific T cells and the production of proinflammatory cytokines (Salama et al., 2003). The overexpression of PD-L1 in APC inhibited immune responses of autoreactive T cells in EAE, while PD-L1-deficient EAE mice showed worse clinical symptoms, evidenced by significantly upregulated IFN-γ, tumor necrosis factor (TNF)-α, and IL-17 (Salama et al., 2003; Zhu et al., 2006; Carter et al., 2007). The release of these inflammatory factors causes severe inflammatory responses, which is considered a critical step in the pathogenesis of MS and EAE (Wagner et al., 2020). The EAE mice exhibited a milder disease severity after treatment with PD-L1 fusion protein for five consecutive days. The therapeutic effects can be attributed to the suppression of Th17 cell development and the inhibition of retinoic acid-related orphan receptor (ROR)-γt and interferon regulatory factor 4 (Herold et al., 2015). Taken together, PD-1/PD-L1 regulates immunosuppressive responses. A better understanding of the regulatory mechanisms of the PD-1/PD-L1 axis in different immune cells may provide valuable information for the identification of novel therapeutic targets for MS/EAE.

## Inhibition of T Cell Proliferation

Lymphocyte proliferation is an early pathogenic step in MS and the inhibition of proinflammatory lymphocyte proliferation represents a therapeutic strategy for MS (Klotz and Eschborn, 2019). PD-1 inhibits the proliferation of T cells by arresting them in the G0/G1 phase (Gonzalez et al., 2005). The blockade of PD-1, however, promotes the proliferation of lymphocytes via upregulating p-AKT during the acute phase of relapsing-remitting multiple sclerosis (RRMS) (Trabattoni et al., 2009). The addition of PD-L1-Ig into T cells significantly inhibited its proliferation *in vitro* by increasing the proportion of cells in the G1 phase, and this effect was reversed when PD-L1 was inhibited (Zhao et al., 2015). Tasuku and colleagues proposed that cell proliferation-related genes are not sensitive to PD-1, and are therefore highly expressed after TCR activation (Shimizu et al., 2020). In the same study, PD-1^–/–^ T cells did not exhibit a hyperproliferative phenotype in response to the stimuli via TCR. Notably, the above effects could be reversed when PD-1 binds to PD-L1, and the efficiency of immunological inhibition depends on the strength of the signal transmitted by TCR and CD28. The inhibitory effect of PD-L1 is significantly enhanced by the blockade of CD28, with TCR-mediated proliferation being reduced to 80% (Freeman et al., 2000). The underlying mechanisms are that the proliferative response is suppressed by the recruitment of Src homology 2 (SH2) domain-containing protein tyrosine phosphatase to transmit negative signals and the inhibition of tyrosine phosphorylation (Freeman et al., 2000). The rat sarcoma (Ras) plays a key role in cell proliferation, differentiation, and survival (Malumbres and Barbacid, 2003; Bertoli et al., 2019), and rapidly accelerates the progression of rat fibrosarcoma (a serine/threonine kinase serving as a downstream mediator of the Ras) (Malumbres and Barbacid, 2003)/MAPK/ERK signaling pathways by promoting cell proliferation and preventing apoptosis (Robbs et al., 2013). Patsoukis et al. (2012a) found that PD-1 selectively inhibited MAPK/ERK activation by inhibiting Ras and subsequent T cell proliferation. PD-1 arrests T cells in the G1 phase by inhibiting the transcription of *SKP2*, a gene that encodes a component of ubiquitin ligase (Patsoukis et al., 2012a). PD-1 suppresses *SKP2* transcription by inhibiting the PI3K/AKT and MAPK/ERK signaling, and inhibiting the formation of the Skp1-Cullin-1-F-box (SCF)^*Skp*2^ ubiquitin ligase complex. SCF^*Skp*2^ can degrade p27^*kip*1^ (a member of the Cip/Kip family) in T cells and suppress the activity of cyclin-dependent kinases (Cdks, promoting cell cycle progression) in all phases of the cell cycle. As PD-1 inhibits the formation of the SCF^*Skp*2^ ubiquitin ligase complex, it contributes to top27^*kip*1^ accumulation and further inhibition of Cdks, which enhances the transcriptional activity and anti-proliferative effect of Smad3 by phosphorylation. Smad3 inhibits cell cycle progression from G1 to S phase, thereby blocking T cell proliferation (Sherr and Roberts, 2004; Patsoukis et al., 2012a; Figure 2). Alternatively, PD-1 can directly repress Smad3 phosphorylation and enhance its anti-proliferative activity. A relatively low level of Smad3 inhibits the anti-proliferative effect of PD-1 (Patsoukis et al., 2012a). Cell division cycle 25 phosphatase (CDC25) is an important regulator of cell cycle transformation and its inhibition leads to cell cycle arrest (Boutros et al., 2007). In addition, the transcription of *CDC25* is increased during T cell activation. PD-1 inhibits T cell proliferation via upregulating the expressions of p15 and p27 and inhibiting CDC25 (mainly CDC25A) (Patsoukis et al., 2012b). The ubiquitin-mediated CDC25A degradation is also markedly upregulated by PD-1 (Figure 2; Patsoukis et al., 2012b). Ultimately, PD-1/PD-L1 transmits an inhibitory signal and decreases the proliferation of T cells to prevent excessive immune responses.

## Mutual Regulation With Cytokines

PD-1/PD-L1 reduces the secretion of proinflammatory cytokines, such as IFN-γ, TNF-α, IL-2, IL-6, and IL-3 to inhibit T cell activation (Freeman et al., 2000; Latchman et al., 2001; Brown et al., 2003). It has been shown that PD-L1^–/–^ mice produce more cytokines, such as IFN-γ, IL-17, and TNF-α (Carter et al., 2007). In the presence of PD-L1, the secretion of IFN-γ by T cells is decreased by 80% and the secretion of IL-2 is also reduced (Freeman et al., 2000). By contrast, PD-1^*High*^ T cells reduce the production of proinflammatory cytokines and increase the expression of anti-inflammatory cytokine IL-10 by upregulating a variety of inhibitory receptors, including T cell immunoglobulin and mucin domain 3, cytotoxic T lymphocyte-associated antigen-4 (CTLA-4), and exhaustion-related transcription factors (e.g., Eomes and Blimp-1) (Ma et al., 2019).

Cytokines are the central component of the PD-1/PD-L1 axis. PD-1 upregulation induced by TCR activation can be significantly inhibited by the blockade of tumor growth factor beta (TGF-β) (Rekik et al., 2015). Furthermore, IFN-γ, TNF-α, IL-6, and IL-17 are inducers of soluble PD-1 (sPD-1) in CD4^+^ T cells (Liu et al., 2015; Bommarito et al., 2017). IL-27 is an anti-inflammatory cytokine that upregulates PD-L1 expression in naïve T cells, and suppresses Th17 cell differentiation and IL-17 secretion through the PD-1/PD-L1 axis (Hirahara et al., 2012). In Waldenstrom macroglobulinemia, the expression of PD-L1 and PD-L2 in T cells was significantly upregulated by IL-21, IFN-γ, and IL-6. IL-21 and IL-6 also induced the secretion of soluble PD-L1 (sPD-L1) *in vitro* (Jalali et al., 2018). IL-12 and IL-6 upregulate the expression of PD-1 following TCR activation by modulating the chromatin structure of the PD-1 (*PDCD1*) gene and promoting *PDCD1* transcription via activation of signal transducer and activator of transcription (STAT)3/STAT4 signaling. However, IL-6 or IL-12 alone fails to exert the above effects (Austin et al., 2014). Notably, the inflammatory microenvironment is likely to impair PD-1/PD-L1-mediated T cell immune suppression. Rheumatoid arthritis and psoriatic arthritis (PsA)-associated inflammatory cytokines, such as TNF-α, IL-6, and IL-1β, have been shown to counteract the suppressive effects of PD-1 on CD4^+^ T cells *in vitro* (Bommarito et al., 2017). Leonard and Lin demonstrated that IL-2, IL-7, and IL-15 bound to their receptors and activated STAT5 phosphorylation (Leonard and Lin, 2000). The activation of STAT5 diminished the PD-1-mediated inhibitory effect (Bennett et al., 2003). Overall, proinflammatory cytokines promote the upregulation of PD-1/PD-L1 and exert PD-1-mediated suppressive effects. Upregulated expressions of PD-1 and PD-L1, in turn, inhibit the production of proinflammatory cytokines and promote the expressions of anti-inflammatory cytokines. A negative feedback between PD-1/PD-L1 and cytokines may exist, which may explain the role of PD-1/PD-L1 as a negative regulator of immune responses.

The expressions of PD-1 and PD-L1 are significantly increased in the brain tissues of MS patients (Ortler et al., 2008), but are downregulated after treatment (Trabattoni et al., 2009). We propose that the activation of a variety of immune cells induces the production of proinflammatory cytokines during the relapsing phase of MS, and then promotes the expression of PD-1/PD-L1. The downregulation of PD-1/PD-L1 after treatment is likely due to significantly reduced proinflammatory cytokines. The effects of secreted inflammatory cytokines on the expression and function of PD-1/PD-L1 in MS remain to be elucidated.

## Regulation of T Cell Differentiation

### Th1 and Th2 Cells

**FIGURE 3:**
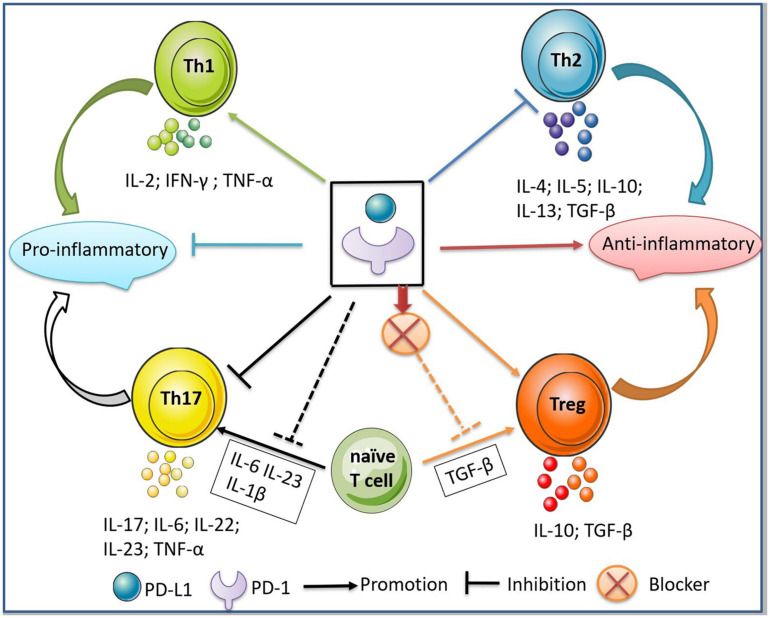
Regulation of PD-1/PD-L1 between Th1/Th2 and Th17/Treg cells. The binding of PD-1/PD-L1 inhibits the differentiation of Th1/Th17 cells and the secretion of pro-inflammatory cytokines, thus further inhibiting the inflammatory responses. Meanwhile, the binding of PD-1/PD-L1 promotes the differentiation of Th2/Treg cells and the secretion of anti-inflammatory cytokines, thus exerting an immunosuppressive effect. In addition, the addition of PD-L1 to the microenvironment where naive T cells differentiate into Th17 cells inhibits the production of Th17, while the blockade of PD-1 to the microenvironment where naive T cells differentiate into Treg inhibits the production of Treg cells.

Th1 cells are a principle subset of effector CD4^+^ T cells that secrete inflammatory cytokines (e.g., IFN-γ, TNF-α, and IL-2) and play a pathogenic role in MS (Olsson, 1995; Sospedra and Martin, 2005). Th2 cells secrete IL-13, IL-4, IL-5, IL-10, and TGF-β to maintain immunological tolerance. A balance between Th1 and Th2 cells is important for human health (Yeh and Lin, 2020). The imbalance between Th1 cell- and Th2 cell-secreted cytokines is a key driver in the pathogenesis of MS (Sospedra and Martin, 2005; Hirahara and Nakayama, 2016). PD-L1 modulates CD4^+^ T cell differentiation by reducing the numbers of IFN-γ^+^CD4^+^ Th1 and IL-17^+^CD4^+^ Th17 cells (Figure 3), while increasing the proportions of IL-4^+^CD4^+^ Th2 and CD4^+^Foxp3^+^ regulatory T (Treg) cells (Ding et al., 2016). In prostate and advanced melanoma cancer, the blockade of PD-1 significantly enhances Th1 effector response by augmenting the secretion of IFN-γ and IL-2, but inhibits Th2 by blocking the production of IL-5 and IL-13 (Dulos et al., 2012; Figure 3). One explanation is that PD-1 inhibition activates the PI3K/AKT and cellular metabolic pathways, promoting the secretion of IFN-γ and IL-2 from T cells. However, another study showed that the blockade of PD-1 had no effect on cytokine secretion by Th2 cells (Li et al., 2015). These inconsistent results may be explained by the Th2-driving conditions in different tumor microenvironments. Additionally, in immune thrombocytopenic purpura, sPD-L1 improves the imbalance between Th1 and Th2 cells by activating the PD-1/PD-L1 pathway, which further inhibits the secretion of IFN-γ and promotes the production of IL-4 and TGF-β (Wu et al., 2019).

Furthermore, the suppression of the inhibitory signal cascade generated by the binding of ITIM, ITSM, and SHP-1/2 to the PD-1 cytoplasmic tail triggers chromatin changes in the promoter regions of the *IL-2* and *IFN-*γ genes and upregulates their transcription (Riley, 2009). In head and neck squamous cell carcinoma, PD-L1 binds to PD-1 to activate the downstream SHP-2, resulting in the inhibition of p-STAT1, T-bet, and p-S6, and the subsequent downregulation of Th1 cytokines, including IFN-γ, TNF-α, and IL-2. However, the secretion of IL-10 from Th2 cells was not affected (Li et al., 2015). The activation of SHP-2 can also bypass the PD-1 signaling to inhibit p-STAT1/T-bet, thereby suppressing immune responses in Th1 cells (Li et al., 2015). Therefore, PD-1/PD-L1 or SHP-2 agonists may serve as promising therapeutic targets for MS.

### Th17 and Treg Cells

Th17 cells represent a subset of CD4^+^ T cells secreting IL-17A, IL-17F, IL-6, IL-22, TNF-α, IL-23, and C-C chemokine receptor 6, which damage the blood-brain barrier (BBB) and enhance its permeability (Littman and Rudensky, 2010; Moser et al., 2020). Furthermore, Th17 cells induce the recruitment of neutrophils, macrophages, and monocytes to the inflamed sites in the CNS (Waisman et al., 2015). Treg cells are a subset of CD4^+^CD25^+^ T cells expressing Foxp3. They have been shown to suppress over-activated immune responses by producing anti-inflammatory cytokines, including TGF-β and IL-10 (Tang and Bluestone, 2008; Lee, 2018). Treg cells also maintain immune homeostasis by inhibiting effector CD4^+^ T cell subsets (Danikowski et al., 2017). Genetic abnormalities of CTLA-4 and CD25, and a low expression level of Foxp3 in Treg cells are closely related to the pathogenesis of MS (Danikowski et al., 2017; Kimura et al., 2018).

The PD-1/PD-L1 interaction plays a vital role in the differentiation, maintenance, and function of Treg cells. There are two main subsets of Treg cells, the “natural” CD4^+^ Treg cells that are developed in the thymus and the “induced” Treg (iTreg) cells that are developed from peripheral CD4^+^ T cells (Haribhai et al., 2016). Previous studies have indicated that the activation of the PI3K/AKT/mTOR signaling pathway strongly inhibits the differentiation and development of iTreg cells. However, PD-1/PD-L1 downregulates p-PI3K, p-mTOR, and p-AKT, and increases the expression of Foxp3 in iTreg cells, thereby promoting iTreg cell differentiation and maintaining their function (Figure 1; Haxhinasto et al., 2008; Francisco et al., 2009; Ding et al., 2016). In contrast, the PI3K/AKT/mTOR pathway positively affects Th17 cell differentiation (Figure 1; Kurebayashi et al., 2012; Nagai et al., 2013). The endolysosomal protease asparaginyl endopeptidase (AEP) is responsible for destabilizing Foxp3 in Treg cells. PD-1/PD-L1 maintains a high expression level of Foxp3 in Treg cells by inhibiting AEP, and therefore generates stable and functionally robust Treg cells *in vivo* and maintains the balance of the immune system (Stathopoulou et al., 2018). sPD-L1 and TGF-β promote the transformation of immature T cells to Treg cells. It has also been reported that the number of Treg cells in PD-L1-deficient mice was significantly decreased compared with the control group (Francisco et al., 2009). *In vivo*, the suppressive effect of Treg cells is reduced when PD-1 is blocked (McGrath and Najafian, 2012).

Many studies have demonstrated that the differentiation between Th17 cells and Treg cells is essential for maintaining the balance of the immune system, and the dysregulation of the system may contribute to MS pathogenesis (Kimura and Kishimoto, 2010; Jamshidian et al., 2013). PD-1/PD-L1 maintains immune homeostasis by inhibiting immune responses of Th17 cells and promoting Treg cells (Figure 3). In a study of pre-eclampsia, the imbalance was manifested by an increase in the number of Th17 cells and a decrease in Treg cells due to low expression of PD-1/PD-L1 at the maternal-fetal interface (Tian et al., 2016). Treatment with PD-L1 Fc promoted the differentiation of naive CD4^+^ T cells to Treg cells *in vitro* (Zhang et al., 2018).

Tumor growth factor beta is a key stimulating factor for the differentiation of Treg cells (Omenetti and Pizarro, 2015). In the presence of TGF-β, PD-1 inhibitors promote Th17 cell responses and prevent the development of Treg cells (Figure 3). In the presence of IL-6, IL-23, and IL-1β, treatment with PD-L1 Fc induces the differentiation of naive CD4^+^ T cells into Treg cells, even though the microenvironment may promote Th17 differentiation (Zhang et al., 2018; Figure 3). These findings indicate that the regulatory effects of PD-1/PD-L1 on maintaining the balance of Th17/Treg are stronger than those of inflammatory factors. The differentiation of naive CD4^+^T cells into Th17 or Treg cells depends on the cellular levels of master regulatory factors, RORγt and Foxp3. When Foxp3 was dominantly expressed, naive CD4^+^ T cells are differentiated into Treg cells, while the excessive expression of RORγt leads to the differentiation of naive CD4^+^ T cells into Th17 cells (Omenetti and Pizarro, 2015). The inhibition of both PD-1 and PD-L1 blocks the PI3K/AKT/m-TOR pathway, enhances the expression of PTEN, and increases the expression of Foxp3 (Figure 1). The blockade of the PI3K pathway regulates the nuclear translocation of RORγt and IL-17, thus reducing Th17 cell differentiation (Francisco et al., 2009; Tian et al., 2016).

The signal transducer and activator of transcription 3 (STAT3) is an essential factor in Th17 cell differentiation and development. STAT3 phosphorylation restrains Treg cell development and IL-10 production (Wei et al., 2008). The aberrant activation of the STAT3 signaling causes an imbalance between Th17 and Treg cells (Wei et al., 2008). A recent study showed that the expression of PD-1 in Th17 cells was significantly upregulated in idiopathic pulmonary fibrosis, which promoted the development of Th17 cells, increased the secretion of IL-17, and therefore created a pro-fibrotic microenvironment (Celada et al., 2018). It has also been confirmed that high expression of PD-1 in Th17 cells enhances the STAT3 signaling, promoting Th17 cell development and IL-17 production (Celada et al., 2018). Both direct and indirect regulatory mechanisms may exist in PD-1-mediated Th17 cell differentiation. PD-1 expressed on Th17 cells can upregulate STAT3 and promote the differentiation and maturation of Th17 cells. In addition, the binding of PD-1 to PD-L1 indirectly decreases the number of Th17 cells by inhibiting the PI3K/AKT pathway. Previous studies mainly focused on the combined regulatory effects of PD-1/PD-L1 on cells. However, PD-1 or PD-L1 alone may also exert regulatory effects on immune cells. The regulatory mechanisms of PD-1 and PD-L1 in immune responses are far more complex than previously understood.

### Paradoxical Role in Tfh Cells

Tfh cells, located at the T cell-B cell border, are a major source of extrinsic factors that promote the formation, expansion, and isotype switching of germinal centers (GCs) (Toellner et al., 1996; Allen et al., 2007). Tfh cells aid B cells and promote neuroinflammation in MS (Schmitt, 2015; Quinn and Axtell, 2018; Huber and Chang, 2020). Tfh cells also promote the infiltration of B cells into the CNS, while Tfh cell deficiency alleviates the symptoms of EAE (Bagaeva et al., 2006; Schafflick and Xu, 2020). Although the regulatory role of PD-1 in Tfh cells has not been fully elucidated, it has been suggested that PD-1/PD-L1 affects the development and function of Tfh cells (Blackburn et al., 2009; Serriari et al., 2010).

Two phenotypes of C-X-C chemokine receptor 5 (CXCR5)^+^ Tfh cells have been identified in both mice and humans: the effector phenotype of CCR7^*lo*^PD-1^*hi*^CXCR5^+^ helper T cells and the resting phenotype of CCR7^*hi*^PD-1^*lo*^CXCR5^+^ helper T cells. Once stimulated with antigen, CCR7^*lo*^PD-1^*hi*^CXCR5^+^ Tfh cells are rapidly differentiated into mature Tfh cells to facilitate humoral immune responses. The Tfh phenotypes with high PD-1 expression are more associated with autoimmune diseases (He et al., 2013). In terms of immunosuppression, the overexpression of PD-1 in Tfh cells reduces the number of CXCR5^*hi*^ or CXCR5^*hi*^Bcl-6^*hi*^ Tfh cells (Shi et al., 2018). In Tfh cells, PD-1 binds to PD-L1 to inhibit the activity of the PI3K signaling pathway, thereby reducing the expression of the follicle guidance receptor CXCR5 and inhibiting the recruitment of Tfh cells to follicles. In addition, PD-1 inhibits the recruitment of Tfh to follicles via recruiting and activating SHP2 through the ITSM motif (Y248) (Shi et al., 2018).

In terms of immune enhancement, PD-1 combines with PD-L1 in the follicular mantle, regulating the chemo-sensing program of CXCR5^+^CCR7^*lo*^S1PR2^+^ Tfh cells (favor localization to the follicular center) and promoting Tfh cells to enter the GC, which is beneficial for B cell maturation and the production of antibodies. PD-1 weakens the potential of Tfh cells to escape from the follicular area by dampening the expression of CXCR3, which further enhances the accumulation of Tfh cells in GC (Shi et al., 2018). High expression of PD-1 on Tfh cells upregulates IL-4 and IL-21, and promotes the maturation of B cells in GCs and plasma cells (Good-Jacobson et al., 2010, 2012). The interaction of PD-1/PD-L1 optimizes B cell affinity maturation, which increases the number of effector cells with a high affinity, and thus prevents the proliferation of autoreactive, low-affinity cells (Good-Jacobson et al., 2010; Shi et al., 2018). High expression of PD-1 on Tfh cells can increase the survival of B cells in GC, which increases the proportion of cells entering into the long-lived B cell compartment and further promotes the generation of long-lived plasma cells (Good-Jacobson et al., 2010). The combination of PD-1 on Tfh cells and PD-L2 on B cells decreases the population of Tfh cells (Figure 3; Peng et al., 2019). PD-1 expressed by Tfh cells inhibits the differentiation of Tfh cells when combined with PD-L1 on DCs (Sage and Schildberg, 2018). One explanation for the regulation of PD-1/PD-L1 expression and Tfh cells is that high PD-1 expression reduces the number of Tfh cells; however, it also enhances the maturation of Tfh cells and the secretion of cytokines. A previous study of Fingolimod-treated MS patients has confirmed that the number of Tfh cells is reduced, but the proportions of PD-1^+^ Tfh cells and activated Tfh cells are increased in this population (Claes et al., 2014). The dual regulatory mechanisms between PD-1/PD-L1 and Tfh cells suggest that PD-1/PD-L1 not only plays an immunosuppressive role but also promotes immune responses. A prevailing hypothesis is that adaptive alteration of PD-1 expression may occur in a chronic inflammatory environment. High PD-1 expression on Tfh cells boosts long-lived antibody responses when the acute cytotoxic response is ineffectual, which protects against pathological damage caused by chronic inflammation (Good-Jacobson et al., 2010).

## Controversial Role of sPD-1/sPD-L1

PD-1/PD-L1 has two forms, the membrane-bound form and the soluble form. The membrane-bound PD-1/PD-L1 is the earliest and the most widely recognized and the sPD-1/sPD-L1 is mainly produced by the proteolytic cleavage of the membrane-bound form (Abu Hejleh et al., 2019). At present, the exact role of sPD-1/sPD-L1 in MS remains vague, and whether immunosuppressive signals, like PD-1/PD-L1, are transmitted is still controversial. Guillain-Barre Syndrome (GBS) occurs in response to T cell-mediated inflammatory autoimmune demyelination of peripheral nerves, leading to an array of symptoms, such as acute weakness of the extremities, hyporeflexia, and areflexia. Experimental autoimmune neuritis (EAN) is a classical animal model of GBS (Shen and Chu, 2018). Ding et al. found that treatment of EAN mice with sPD-L1 alleviated the symptoms of EAN, inhibited inflammatory cell infiltration and sciatic nerve demyelination, and delayed neurological progression of EAN (Ding et al., 2016). Mahoney and Shukla (2019) discovered an alternative splicing variant of the *PD-L1* gene, which encodes the secreted form of PD-L1 (secPD-L1), consisting of 18 amino acid tail sequences that contain cysteine and can therefore be homo-dimerized (Mahoney and Shukla, 2019). The secPD-L1 is functionally active, and thus can bypass the binding to PD-1 and independently inhibit T cell proliferation and IFN-γ production stimulated by CD3/CD28 co-activation. The inhibitory effect of secPD-L1 is stronger than that of the extracellular domain of monomeric sPD-L1. They also found that the homodimers of secPD-L1 are expressed in both normal tissues and activated myeloid cells that are converted from peripheral blood cells and tumors (Mahoney and Shukla, 2019). The data on the potential role of sPD-1/sPD-L1 in MS is virtually non-existent. Notably, the injection of EAE mice with B7-H1-Ig fusion protein effectively alleviated the clinical symptoms of EAE (Herold et al., 2015). These data implies that sPD-L1 may maintain immune homeostasis in MS by negatively regulating immune responses. Nevertheless, some controversies remain. It has been reported that sPD-1 was upregulated in the inflammatory microenvironment in RA and PsA, which counteracted PD-1-mediated suppression of CD4^+^ T cells and increased the proliferation of effector T cells (Bommarito et al., 2017). Collectively, the sPD-1 receptor may modulate PD-1 ligation under physiological conditions, which increases the resistance of CD4^+^ T cells to PD-1-mediated suppression. Also, the increased secretion of sPD-1 in RA and PsA is mainly attributed to the elevated secretion of inflammatory cytokines, such as TNF-α and IL-6 (Bommarito et al., 2017). Similar to RA, sPD-1 inhibits the PD-1/PD-L1 axis and enhances the activity and proliferation of T cells in aplastic anemia which involves another splicing variant of *PD-1*, deltaex3. This variant lacks a transmembrane domain but has a soluble extracellular domain, which functionally blocks the regulation of the membrane-bound form of PD-1 on activated T cells, thus counteracting the inhibition effect of PD-1 on T cells (Dong et al., 2003; Wan et al., 2006; Wu et al., 2009). The level of sPD-1 is significantly increased in patients with autoimmune hepatitis (AIH) during the period of disease activity and in AIH patients who have poor response to standard treatments, which suggests that sPD-1 exerts a proinflammatory effect and can be used as a clinical biomarker to evaluate the progression of AIH (Hadley et al., 2020).

The role of sPD-1/sPD-L1 in the immune response is controversial. The soluble forms of PD-1 and PD-L1 increase the complexity and diversity of the PD-1/PD-L1 pathway. The regulatory effects of sPD-1/sPD-L1 in autoimmune diseases and tumors have been reported in previous studies (Table 1). They found that the expression of sPD-1/sPD-L1 was upregulated in patients with autoimmune diseases compared with healthy subjects, but downregulated after treatment, suggesting that sPD-1 may resist the inhibitory effect of membrane-bound PD-1 on T cells, thereby indirectly enhancing immune responses (Wu et al., 2009; Shi et al., 2013; Liu et al., 2015; Aarslev et al., 2017; Yan et al., 2019). It is also likely that the upregulation of sPD-1 during severe inflammation is a self-protection mechanism of the body to combat inflammation. *In vitro*, sPD-L1 inhibited T cell activation, proliferation, and metabolism, thereby promoting tumor immune escape. High sPD-1 levels may be associated with the exhaustion of T cells and can be used as a prognostic marker of cancers (Zhou et al., 2017; Jalali et al., 2018). Moreover, high expression of sPD-L1 in tumors is related to the splicing variant of the *PD-L1* gene, which lacks the transmembrane domain and can cause abnormal secretion of sPD-L1 (Zhou et al., 2017). The splicing variation may be related to genetic predisposition. We speculate that the polymorphism of the *PD-L1* gene may also be affected by pathological microenvironment. Cytokines, chemokines, and even exosomes in the microenvironment may cause abnormal secretion of sPD-1/sPD-L1. The contradictory role of sPD-1/sPD-L1 in autoimmune diseases and tumors suggest that they may be secreted by different cells. Tumor cells are the main source of sPD-1/sPD-L1 in tumors, while in autoimmune diseases, sPD-1/sPD-L1 are mainly produced by immune cells. Therefore, there might be different subtypes of sPD-1/sPD-L1 with various functions. Proteomics, metabolomics, or transcriptomics may be used to identify the subtypes of sPD-1/sPD-L1 for targeted therapies.

**TABLE 1:** The studies of sPD-1 and sPD-L1 in autoimmune diseases and tumors.

Variable (sPD-1 or sPD-L1)	Diseases (year) (reference number)	Specimens	Groups	Results	The factors affecting sPD-1 or sPD-L1 expression	The regulatory effects of sPD-1 or sPD-L1	Conclusion
sPD-1	Rheumatoid arthritis (RA) (2015) (Liu et al., 2015)	Sera, synovial fluid	Osteoarthritis (OA); Healthy control (HC); The level of RA disease activity (low, moderate, high)	RA > OA RA > HC Hi-RA > Lo-RA	IFN-γ, TNF-α, IL-17A upregulated sPD-1	sPD-1 blocked the inhibitory effect of membrane-bound PD-1 on T cell activation, and induced the Th1/Th17 immune response	sPD-1 aggravated the severity of RA
sPD-1 sPD-L1	Myasthenia gravis (MG) (2019) (Yan et al., 2019)	Plasma	Untreated-stage MG (USMG); Remission-stage MG (RSMG); HC	sPD-1: USMG > RSMG USMG > HC sPD-L1: no difference	The level of sPD-1 was positively correlated with the number of Tfh cells and IL-21, and had no correlation with the expression of PD-1 on CD4^+^ T cells	sPD-1 may interfere with the immunosuppressive effect of PD-1 on Tfh cells, thereby over-activating Tfh cells	The level of sPD-1 was positively correlated with the concentration of acetylcholine receptor antibody, which might promoted immune response and aggravated the progression of MG
sPD-1	Autoimmune hepatitis (AIH) (2017) (Aarslev et al., 2017)	Plasma	Active disease; Standard-therapy: complete responders, Incomplete-responders; HC	Active disease and incomplete-responders > responders and HC	sPD-1 was produced by the release of activated PD-1^+^ T cells	sPD-1 promotes the activation and development of T cells by blocking the binding of PD-L1 on monocytes and PD-1 on T cells	The level of sPD-1 can reflect the degree of T cell activation, which promotes immune response and aggravates AIH
sPD-L1	Type 2 diabetes mellitus (T2DM) (2013) (Shi et al., 2013)	Serum	T2DM; T2DM with atherosclerotic (AS); HC	T2DM > HC T2DM with AS > T2DM	The amount of sPD-L1 was positively correlated with IFN-γ	sPD-L1 enhances T cell activation, proliferation, and development by blocking PD-1/PD-L1 signaling	Elevated sPD-L1 is closely associated with the severity of diabetic atherosclerotic macrovascular disease
sPD-1	Aplastic anemia (AA) (2009) (Wu et al., 2009)	Sera	AA; severe AA; Chronic AA; HC	AA > HC Severe AA > chronic AA	The over-expression of PD-1ΔEx3 (a gene polymorphism of PD-1) increased the yield of sPD-1	sPD-1 may block the immunosuppressive function of PD-1 ligands by acting as the autoantibody of PD-1 ligands, thereby inhibiting T cell apoptosis, and enhancing T cell proliferation and T cell toxicity	sPD-1 promotes the immune response and aggravates AA
sPD-1	Human immunodeficiency virus (HIV) (2019) (Zilber et al., 2019)	Plasma	Primary HIV infection; HC	HIV > HC After treatment: HIV = HC	sPD-1 was positively correlated with the expression of PD-1 on the surface of CD4^+^ and CD8^+^ T cells	sPD-1 is associated with T cell exhaustion and can reflect the degree of T cell exhaustion	sPD-1 can act as a measure biomarker of immune exhaustion in HIV
sPD-L1	Waldenstrom macroglobulinemia (WM) (2018) (Jalali et al., 2018)	Serum	WM; HC	WM > HC	IL-21 and IL-6 increased sPD-L1 secretion in the culture medium of WM cell lines	The proliferation of T cells is inhibited by sPD-L1 by down-regulating p-AKT, p-ERK and the cell cycle protein cyclin A. sPD-L1 also regulates the metabolic rate of T cells by reducing the maximum respiration capacity of T cells and inhibiting the production of mitochondrial adenosine triphosphate	sPD-L1 promotes disease progression in WM.
sPD-L1	Malignant melanoma (MM) (2017) (Zhou et al., 2017)	Plasma, sera	MM: pretreatment; HC	Pretreatment > HC	IFN-γ, IFN-α, and TNF-α induced the secretion of sPD-L1	sPD-L1 inhibits the activation and proliferation of T cell	High pretreatment level of sPD-L1 is closely related to the poor effect of PD-1 blockade
sPD-1 sPD-L1	Diffuse large B-cell lymphoma (DLBCL) (2021) (Vajavaara and Mortensen, 2021)	Serum	Prior to treatment, After three immunochemotherapy courses, At the end of therapy	Prior to treatment > after three therapy courses and at the end of therapy	The sPD-1 level of prior to treatment was positively correlated with the number of PD-1^+^ T cells and translated to a lower survival	Pretreatment sPD-1 level reflects T cell exhaustion	High expression of sPD-1 decreases survival of patients. sPD-L1 is not associated with poor outcome

The immunomodulation of sPD-1/sPD-L1 in MS has not been fully characterized. Several questions need to be addressed in future studies: (1) What are the plasma levels of sPD-1/sPD-L1 in MS patients, especially in acute and remission phases? (2) Is the immune response in antigen-specific T cells positively or negatively regulated in MS? (3) Does the immune response affect PD-1 expression in T cells and PD-L1 expression in DCs? (4) Is it possible to exert an immunosuppressive effect on T cells and DCs by regulating sPD-1/sPD-L1?

## Therapeutic Agents Targeting PD-1/PD-L1

Multiple sclerosis is generally considered incurable. Current treatment strategies aim to: (1) reduce immune-inflammation in the acute phase, therefore alleviating the symptoms; (2) reduce the recurrence of MS using the “disease-modifying” therapies (DMTs). Glucocorticoids are widely recognized as the first-line treatment for MS in the acute phase. Dexamethasone has been found to upregulate the expression of PD-1 in T cells in cancer therapy (Xing et al., 2015). With the widespread use of PD-1 antibodies in tumor therapy, potential side effects have also been reported. For example, the function of the adrenal cortex may be impaired after PD-1 antibody treatment, leading to adrenocortical insufficiency and decreased cortisol secretion, suggesting a correlation between the PD-1/PD-L1 axis and glucocorticoids. Although it remains unclear whether glucocorticoid therapy affects the expression of PD-1/PD-L1 in immune cells in MS, we propose that glucocorticoid therapy may exert an immunosuppressive effect by increasing the expression of PD-1 on immune cells. Estrogen plays an immunoprotective role in EAE mice by upregulating PD-L1 on monocytes and macrophages in the peripheral blood and CNS (Seifert et al., 2019). At the same time, estrogen increases the expression of PD-L1 on DCs, which further promotes the formation of tolerant DCs and therefore alleviates the development of EAE (Papenfuss et al., 2011). Estrogen also protects against EAE by upregulating PD-L1 on B cells (Bodhankar et al., 2011) and Treg cells (Wang et al., 2009).

The DMTs, including IFN-β, fingolimod, siponimod, teriflunomide, natalizumab, rituximab, ocrelizumab, and dimethyl fumarate, effectively reduce the recurrence in patients with MS (Bar-Or et al., 2014; Ciotti and Cross, 2018; Rae-Grant et al., 2018). Short-term IFN-β treatment has been shown to upregulate the expression of PD-L1 on monocytes in MS patients (Feng et al., 2019). IFN-β also alleviates MS by upregulating PD-L1 on semi-mature DCs and inhibiting the activation of CD4^+^ T cells, which maintains peripheral immune tolerance (Schreiner et al., 2004). Additionally, IFN-β treatment directly promotes the expression of PD-L1 on CD4^+^ T cells and exerts an immunosuppressive effect on T cells in MS patients (Wiesemann et al., 2008). Fingolimod (FTY720) is an immunomodulatory drug for the treatment of RRMS. It is the first-generation sphingosine-1-phosphate receptor (S1PR) that reduces the frequency of disease recurrence in MS patients by restraining lymphocytes in lymph nodes and preventing further damage to the CNS (Brinkmann et al., 2010; Cohen et al., 2010; Claes et al., 2014). The expression of PD-1 on Tfh cells is evidently increased after fingolimod treatment (Claes et al., 2014). Preclinical studies showed that co-culture of FTY720-treated bone marrow-derived dendritic cells and T cells promoted the proliferation of Treg cells and upregulated PD-1 expression on T cells (Heng et al., 2010). Siponimod is the second-generation S1PR that has been approved to treat secondary progressive MS (Mao-Draayer et al., 2017). Siponimod upregulates the expression of PD-1 on Treg cells and promotes the proliferation of Treg cells in MS (Wu et al., 2020).

The *PLP1* mutant was first identified in MS patients with neuroinflammation, axonal degeneration, neuron loss, and brain atrophy (Warshawsky et al., 2005; Groh et al., 2016). The mutation of the *PLP1* gene leads to myelin sheath and axonal damage by enhancing the immune effect of cytotoxic CD8^+^ T lymphocytes (Groh et al., 2016). Mice with mutant *PLP1* gene showed a reduced number of CD8^+^ effector T cells in the CNS and increased proliferation of CD8^+^CD122^+^PD-1^+^ regulatory T cells after treatment with teriflunomide (Groh et al., 2018). It remains unclear whether teriflunomide affects the expression of PD-1 on CD4^+^ T cells or the expression of PD-L1 on APC in MS patients. Dimethyl fumarate, an immunomodulator approved for the treatment of RRMS, inactivates cysteine-rich proteins by succination (Blair, 2019; Saidu et al., 2019). Dimethyl fumarate inhibits aerobic glycolysis in activated myeloid and lymphocytes by succinating the glycolytic enzyme glyceraldehyde 3-phosphate dehydrogenase, thereby exerting an anti-inflammatory effect and preventing recurrence (Kornberg and Bhargava, 2018). In one MS patient, PD-1 was highly expressed on CD8^+^ T cells and memory effect T cells after dimethyl fumarate treatment, which promoted the exhaustion of T cells, while the PD-1 level was decreased after discontinuation of dimethyl fumarate (Garcia et al., 2020).

In summary, DMT plays an immunoregulatory role via regulating the expression of PD-1/PD-L1 on different immune cells. It is worth noting that reports in this field are scarce and future studies using animal models are warranted.

## Recent Research in MS

Single-nucleotide polymorphisms (SNPs) have been a focus of interest for several years. Studies have shown that SNPs in *PDCD1* are associated with a number of autoimmune diseases, including SLE (Fathi et al., 2020), RA (Kong et al., 2005), T1D (Qian et al., 2018), and ankylosing spondylitis (AS) (Liu et al., 2011). To date, only two studies have reported the association between SNPs in *PDCD1* and MS (Kroner et al., 2005; Pawlak-Adamska et al., 2017). One study performed genotyping analysis of PD-1 expressed on T cells from patients with different types of MS. It was found that an intronic 7146G/A polymorphism of PD-1 had no effect on MS susceptibility, but significantly promoted the progression of MS. The functional assessment of the protein encoded by the mutated *PD-1* gene showed that the secretion of IFN-γ by T cells was decreased (Kroner et al., 2005). Although this study did not investigate the effect of the mutated *PD-1* gene on T cell proliferation, apoptosis, or differentiation, it highlighted the potential involvement of the mutation of the *PD-1* gene in the pathogenesis of MS. Another study showed that the polymorphism in *PDCD1* affected the symptoms and severity of RRMS (Pawlak-Adamska et al., 2017). The authors selected three SNPs of PD-1: PD-1.3 (rs11568821, intron 4), PD-1.5 (rs2227981, exon 5), and PD-1.9 (rs2227982, exon 5). They found that the presence of the PD-1.5T allele was associated with the initial presentation of MS. For example, it mainly caused pyramidal tract symptoms but prevented diplopia. Moreover, the PD-1.3G/PD-1.5T/PD-1.9T haplotype did not lead to severe RRMS, whereas ePD-1.3G/PD-1.5T/PD-1.9C haplotype prevented diplopia (Pawlak-Adamska et al., 2017). As for the mechanism of action of PD-1.3A, Prokunina et al. (2002) showed that PD-1.3A destroyed the binding site in Runt-related transcription factor 1, reduced the mRNA expression of *PD-1*, inhibited autoimmune tolerance, and induced autoimmune reactions. Regarding the mechanism of action of other SNPs in *PD-1*, different haplotypes may lead to different clinical symptoms or types of MS, which require further investigation. Previous literature described that the genotype frequency of the PD-1.3 site varied greatly among different ethnic groups (Prokunina et al., 2002). Therefore, independent analysis in a targeted manner is needed to identify the relationship between SNPs in *PD-1* and MS. Ethnic and regional differences should also be taken into account.

MicroRNAs (miRNAs) are small endogenous non-coding RNA molecules composed of approximately 21–25 nucleotides (Kim et al., 2017). They usually target one or more mRNAs for degradation or regulate the translation of targeted mRNAs (Chavali et al., 2011). Nearly 30% of protein-coding gene expression is controlled by miRNAs (Kim et al., 2017). They play a diverse role in regulating cell growth, development, proliferation, differentiation, maturation, and apoptosis (Roccaro et al., 2009), and mediate many physiological and pathological processes at the posttranscriptional level (Ferrajoli et al., 2013). Recent studies have demonstrated that miRNA is involved in the pathogenesis of MS and can be used as a biomarker for the diagnosis and prognosis of MS, as well as for monitoring disease activity (Piket et al., 2019; Mansoor et al., 2020). More than 500 miRNAs are dysregulated in immune cells, cerebrospinal fluid, and serum samples from MS patients (Mansoor et al., 2020). Small-molecule inhibitors for specific miRNAs or antisense oligonucleotides may be used to treat MS in the future (Tufekci et al., 2010; Zare-Shahabadi et al., 2013).

In MS patients who failed to respond to first-line therapy, the expression of miR-155, a proinflammatory miRNA, is significantly upregulated on T cells (Arruda et al., 2015). MiR-155 deletion in EAE mice suppressed the secretion of IFN-γ and IL-17, inhibited the development of Th17 cells, and reduced oxidative stress response and BBB permeability (Juźwik et al., 2019). MiR-16 and miR-142-3p also play a proinflammatory role in MS via regulating T cell activation. Their overexpression is related to T cell-mediated autoimmune inflammation (Arruda et al., 2015). It was found that miR-16, miR-155, and miR-142-3p on T cells were significantly upregulated in MS patients who failed to respond to first-line therapy, while the expressions of *FoxP3*, *FoxO1*, *PDCD1*, and *IRF2BP2* were notably decreased. After autologous hematopoietic stem cell transplant (AHSCT), the expressions of miR-16, miR-155, and miR-142-3p were decreased, while the levels of *FoxP3*, *FoxO1*, *PDCD1*, and *IRF2BP2* were significantly elevated. The upregulation of *PDCD1* after AHSCT may be due to the downregulation of miR-16, since *PDCD1* is a target gene of miR-16 (Arruda et al., 2015). We speculate that miR-155 may also be involved in *PDCD1* expression. MiR-155^–/–^ mice were highly resistant to EAE, while PD-1 deletion restored their susceptibility to EAE (Zhang and Braun, 2014). The mechanism may be related to the antagonistic regulation of miR-155 and PD-1 on suppressor of cytokine signaling 1 (SOCS1) and its downstream STAT1/3 pathway, which stimulates the production of IFN-γ and IL-17 (Zhang and Braun, 2014). Another study showed that miR-155 depletion inhibited the activation of T cells, but this effect could be reversed by blocking PD-1 in tumors (Huffaker et al., 2017). The PD-1 antibody therapy increases the expression of miR-155 on T cells in tumors (Martinez-Usatorre et al., 2019). The Bortezomib therapy increased the expression of miR-155 in T cells, thereby downregulating the levels of its targets, SOCS1 and SH2-containing inositol 5′-polyphosphates 1 (SHIP1), and significantly decreasing the number of SHIP1^+^PD-1^+^ T cells (Renrick et al., 2021). Furthermore, miR-155 suppressed tumor progression by inhibiting the expression of PD-L1 in tumors (Yee et al., 2017).

The recurrence rate of pregnant MS patients is 66% less than that of other MS patients. One study showed that the expressions of miR-1, miR-20a, miR-28, miR-95, miR-146a, miR-335, and miR-625 in the PBMC of pregnant MS patients were downregulated, while the levels of *IL10*, *PDL1*, and *PDL2* were increased compared with untreated MS patients. They concluded that *IL10*, *PDL1*, and *PDL2* were the targets of these miRNAs and there was a negative correlation between them (Søndergaard et al., 2020). The regulatory mechanism requires further exploration.

Mice with miR-21 deficiency showed Th17 differentiation defects and were tolerant to EAE (Carter et al., 2012; Murugaiyan et al., 2015). MiR-21 suppressed the expression of PTEN, leading to the activation of the PI3K/AKT and MAPK/ERK pathways (Meng et al., 2007; Bao et al., 2013). The activation of the PI3K/AKT and MAPK/ERK pathways increased T cell proliferation and differentiation (Haxhinasto et al., 2008; Patsoukis et al., 2012a). Zheng et al. (2019) found that the expression of miR-21 in patients with gastric cancer resection was decreased, which affected the differentiation of T cells by upregulating PD-1/PD-L1. Low miR-21 expression resulted in an imbalance of Th17/Treg cells (an increase in the number of Treg cells and a decrease in the number of Th17 cells). The upregulation of miR-21 increased the number of Th17 cells (Zheng et al., 2019). Many miRNAs are involved in the pathogenesis of MS. Previous data has confirmed a tight connection between miRNAs and PD-1/PD-L1, suggesting that miRNAs alter immune function by regulating the PD-1/PD-L1 axis (Ashrafizadeh et al., 2020). Taken together, miRNAs regulate the expression of PD-1/PD-L1 and may be used as therapeutic targets for MS.

## Concluding Remarks and Future Directions

The upregulation of PD-1/PD-L1 in tumor microenvironment leads to impaired immune cell function and premature apoptosis. Autoimmune diseases are caused by excessive immune responses, resulting in damage to normal tissues. The PD-1/PD-L1 signaling pathway may be dysregulated in MS [e.g., the dysregulated PD-1 axis in AIH (Aarslev et al., 2017)]. The loss of immune homeostasis leads to a robust immune response that cannot be controlled properly. The loss of immune homeostasis in MS may be related to genetic interactions, environmental factors, dietary factors, or other diseases. The PD-1 receptor needs to bind to its ligand in order to suppress the effector immune response. Therefore, two reasons may explain the dysregulation of the PD-1/PD-L1 signaling pathway. Firstly, the number of immune cells increases sharply, but the upregulation of PD-L1 is a slow process. Secondly, the expression PD-1 on cells may be impaired under the stimulation of cytokines or Ag receptors, leading to excessive immune responses. Future investigations should focus on whether the increase in the amount of PD-L1 and/or the upregulation of PD-1 on immune cells can restore immune homeostasis in MS.

In future studies, whether the expression of PD-1/PD-L1 in the immune cells of MS patients is lower than that of patients with non-autoimmune diseases can be detected using flow cytometry or polymerase chain reaction. The expression of PD-1/PD-L1 in MS patients at different stages and following different treatments can also be compared. Furthermore, it is necessary to assess the levels of sPD-1/sPD-L1 in MS patients and to determine whether they play a proinflammatory or anti-inflammatory role in MS and whether they may cause tolerance or resistance of T cells to the PD-1 pathway.

Whether the PD-1 pathway fails to exert an immunosuppressive effect in MS also warrants investigation. Attention should be paid to the selection of healthy subjects as the control group, since the PD-1/PD-L1 pathway is only activated when immune cells are activated. It may be more appropriate to compare MS patients at different stages. Immune cell extraction and *in vitro* cell culture may also be performed and genetic testing can be used to explore whether the mutation on *PD-1/PD-L1* exists in MS. It is also worthy to explore whether the transcription and translation of the mutant gene, and the post-translational modification and the immune function of the synthetic PD-1/PD-L1 proteins are altered in MS. Moreover, transcriptomics, proteomics, and metabolomics can be used to assess functional changes of immune cells expressing PD-1/PD-L1 in MS patients.

Should future studies suggest that the PD-1 pathway is changed in MS, molecules that can alter the levels of PD-1/PD-L1 (such as sPD-L1) may be used to regulate the immunosuppressive function of the PD-1 pathway in MS. In addition, the downstream targets of the PD-1/PD-L1 pathway (such as SHP2 and PTEN agonists, PI3K/AKT/mTOR inhibitors, etc.) may also be potential therapeutic targets for MS. However, drugs that inhibit the proliferation and differentiation of immunomodulatory immune cells may not be beneficial to the regeneration and recovery of myelin. Gene therapy may also be used to regulate the PD-1/PD-L1 pathway in immune cells. However, whether high PD-1/PD-L1 expression will increase carcinogenic risk should be investigated. The anti-PD-1 antibody has been found to induce autoimmune diseases in cancer treatment (Naidoo et al., 2016; Sibaud, 2018) and therefore might also exert toxic effects in MS patients.

## Author Contributions

HL drafted the manuscript. CZ, JH, and SL helped to edit the manuscript. TJ and JZ performed the critical revisions. All authors read and approved the final manuscript.

## Conflict of Interest

The authors declare that the research was conducted in the absence of any commercial or financial relationships that could be construed as a potential conflict of interest.

## Publisher’s Note

All claims expressed in this article are solely those of the authors and do not necessarily represent those of their affiliated organizations, or those of the publisher, the editors and the reviewers. Any product that may be evaluated in this article, or claim that may be made by its manufacturer, is not guaranteed or endorsed by the publisher.
